# Comparative study between UVB 313 nm, UVC 254 nm, and far UVC 222 nm light on the aging of polyamide 66

**DOI:** 10.1016/j.heliyon.2024.e39415

**Published:** 2024-10-18

**Authors:** Abel Hurtado Macias, M. Román-Aguirre, R.P. Talamantes, Karen M. Soto, José Luis Reyes Araiza, Nestor Méndez-Lozano, Miguel Apátiga-Castro, Jorge Pineda-Piñón, José Ramon Gasca Tirado, José M. López-Romero, A. Manzano-Ramírez

**Affiliations:** a Department of Metallurgy and Structural Integrity, National Nanotechnology Laboratory Centro de Investigación en Materiales Avanzados S.C, Chihuahua, Chihuahua Mexico; b Department of Engineering and Chemistry of Materials, National Nanotechnology Laboratory Centro de Investigación en Materiales Avanzados S.C, Chihuahua, Chihuahua Mexico; c Centro de Investigaciones y de Estudios Avanzados Del I.P.N. Unidad Querétaro, Querétaro, Querétaro, C.P. 76230, Mexico; d Facultad de Ingeniería, Universidad Autónoma de Querétaro, Centro Universitario, Cerro de Las Campanas S/n, C.P. 76010, Querétaro, Querétaro, Mexico; e Universidad Del Valle de México, Campus Querétaro, Blvd. Juriquilla no. 1000 A Del. Santa Rosa Jáuregui, C.P. 76230, Querétaro, Qro., Mexico; f Centro de Física Aplicada y Tecnología Avanzada, Universidad Nacional Autónoma de México, A.P. 1-1010, C.P. 76000, Querétaro, Qro., Mexico; g Instituto Politécnico Nacional, Centro de Investigación en Ciencia Aplicada y Tecnología Avanzada, Unidad Querétaro, Cerro Blanco No. 141, Colinas Del Cimatario, Querétaro, 76090, Qro, Mexico; h Departamento de Ingeniería, Universidad de Guanajuato. Guanajuato, C.P. 36000, Mexico

**Keywords:** COVID-19, Polymeric materials, Aeronautical industry, UVC light, Disinfection, Nanoindentation

## Abstract

Polyamide-66 underwent substantial growth worldwide in the late 1930′s. It can be found in several public spaces. After 2019, due to the coronavirus disease (COVID-19), the sanitization of the interior of aircraft and public spaces by using UV light became important. Most of all, the effect of the far UVC 222 nm became a research hot spot and is still a research blank. In the present work**,** a comparative study on the post-exposure damage of polyamide- 66 occurred when controlled ultraviolet irradiation (UVC 254 nm, far UVC 222 nm, UVB 310 nm) and moisture-condensation were carried out. Chemical (FTIR), thermal (DMA), and nano-mechanical properties were evaluated. FTIR analysis showed the formation of O-H and/or N-H bonds since there appeared absorptions at 3400 cm^−1^ along with the vanishing of other signals related to C-N bonding. These changes are more evident in samples exposed to UVC 254 nm followed by samples that were irradiated with UVC 222 nm. Samples that were aged with UVA 313 nm didn't show a change in FTIR spectra. FTIR on spectra and nanoindentation showed that UVB 313 nm produced a lower aging effect on this material. In contrast, UVC 254 nm light caused the highest degree of surface chemical-mechanical change attributed to cleavage/crosslinking reactions initiated by free radicals produced by UV light and moisture. On the contrary, far UVC 222 nm light presented moderate effects on Polyamide-66. Glass-transition temperature (Tg) diminished as the time to exposure increased attributed to water absorption and surface damage, being the highest change at −16.05 °C for samples irradiated with 245 UVC nm. However, the potential **of** far UVC 222 nm light for COVID-19 sanitization**,** with no significant degradation effects on polymeric materials**,** is a promising finding that should be further explored and **could provide a hopeful solution in the fight against the pandemic.**

## Highlights

• Far UVC 222 nm irradiation caused moderated affectation on polymers.
• UVB 313 nm irradiation produced an aging effect that cannot be detected by FTIR.
• UVC 254 nm light caused the highest affectation on polymers.
• Far UVC 222 nm can be used to inactivate coronavirus in publicly occupied spaces.

## Graphical abstract

**Figure:**
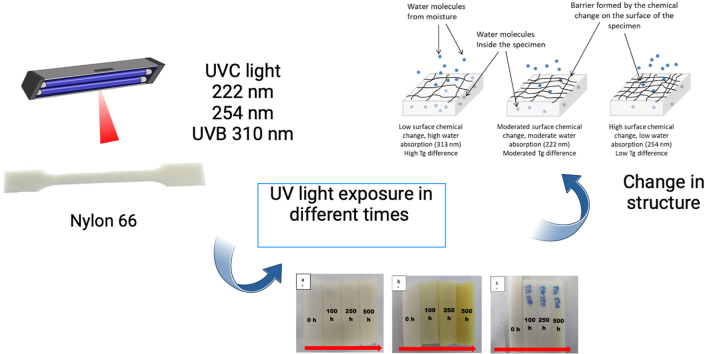

## 1 Introduction

Polyamides, commonly known as nylons, are some of the most commercially important engineering polymers. These materials, which were first introduced as aliphatic polyamides or nylons, were a groundbreaking industrial innovation. Their use has steadily grown worldwide since their invention by Wallace Carothers in 1935 at the Du Pont Company [1,2]. A key feature of nylons is their robust interchain interaction, which stems from the hydrogen bonding between amide groups (–CONH–) on adjacent chains, as well as dipole–dipole interactions [1,2].

An amide group (–CONH–) is, in its molecular structure, the product of a condensation reaction between a diamide and a diacid or a diacyl halogenide [3,4]. This categorizes amide groups into two main types: aliphatic and aromatic polyamides [2]. Nylon refers to a generic class of synthetic polyamides derived mainly from aliphatic monomers [4] and are some of the most commercially important engineering polymers [5]. The chemical characteristics may be determined by the functional end groups (amino group - NH2 and carboxylic acid - COOH) and chain amide groups (-CONH-) present [4]. Polyamides or nylons are considered high-performance plastics. They exhibit high temperature and electrical resistance. They find their use in the automotive, transportation, consumer goods, and electrical and electronics (E&E) industries. They have also found their most extensive application range in tires, carpets, stockings, upholstery, and adhesives [2]. Due to its superb balance of strength, ductility, and heat resistance, it is popular in every significant market using thermoplastic materials. Dubois, Philippea et al., 2009 and Kyulavska, M. et al., 2019 handbooks [6,7], provide an excellent guide to PA6, PA66, PA11, and PA12 Variants. However, it is crucial to know that nylon may undergo degradation when exposed to UV irradiation from sunlight or artificial light sources [[8], [9], [10], [11]]. Stowe et al. reported that free radicals are responsible for chain scission at the methylene group adjacent to the nitrogen [12]. However, Grayson and Wolf [13] and Lock and Frank [8] stated that the chain scission in nylon 6.6 occurs at the amide linkage.

The World Health Organization (WHO) declared a global pandemic in March 2020; the first report was in Wuhan, China, in December 2019. After that, the virus (COVID-19 was detected in more than 200 countries, and there was actually an exponential increase in the number of infections because of the appearance of new variants [14,15]. In human beings, the transmission of the virus is human-to-human through respiratory droplets and contact with surfaces of infected aerosol [16]. Nowadays, several vaccines have been developed and approved for general or emergency use worldwide, leading to the immunization of a critical mass of the world's population as one way to control the pandemic. However, global immunization has not been achieved due to several local factors and new varieties of Sars-CoV2. Airlines have adopted various measures, such as social distancing, shorter boarding times, and surface and air disinfection [17].

Disinfectants are a quick control method for inactivating coronaviruses, including SARS-CoV-2. S.M. Sharafi et al. [16] identified five groups of disinfectants: chlorine-containing disinfectants, alcohol, UV irradiation, Hydrogen peroxide, and other disinfectants, each effective in different environments against SARS-CoV-2. Povidone-iodine or chlorhexidine is recommended when there is a risk of SARS-CoV-2 contamination, especially for open wounds [17]. In contrast, the efficacy of several povidone-iodine (PVP-I) products could help destroy the virus in other settings [18]. However, it is important to interpret research results cautiously, as the reported inactivation effectiveness varies depending on the laboratory conditions. For instance, residues of virus on laboratory benches can be inactivated by UV irradiation. However, the inactivation potential was low (presumably the UV lamp used was a UVB since it was irradiated under normal biosafety cabinet UV lights) [18], but when spraying and wiping the bench with 70 % ethanol, followed by UV irradiation, the test results showed complete inactivation of the virus [18]. On the contrary, inactivation occurred more efficiently when UVC light was used [19]. There are physical sources to achieve virus inactivation, like UV irradiation and temperature [16], which have been confirmed effective in inactivating the virus since the former can be absorbed by nucleic acids, inducing the formation of covalent bonds between adjacent pyrimidines, which, in the case of viruses, would affect the replication machinery [19]. The electromagnetic radiation bands of UVC and UVB are considered operative [20]. It is important to be aware that UV irradiation can be produced or enhanced using several materials like nitrides, sulfides, and metal oxides [21,22], and detected by using Nanostructured materials in photodetector sensors [23]. Direct exposure to conventional germicidal UVC lamps (254 nm) in occupied public spaces is not desirable due to the well-known harmful effects caused to the eyes and skin in humans [[24], [25], [26]] and polymeric materials [[27], [28], [29], [30]].

On the contrary, Far UV-C emitting devices (ranging from 200 to 230 nm), have shown their utility and biological impacts [24]. However, Chikako and co-workers indicate that studies on the biological effects of these Far UVC devices must be motivated to understand and help balance their usefulness and risk. In addition, it has a minimum penetration depth of a few microns. This limited ability to penetrate biological materials is still more significant than the size of viruses and bacteria and contrasts with conventional germicidal UV light, 254 nm (or higher) [31,32]. However, this penetration is still more significant for viruses and bacteria sizes. In addition, far ultraviolet light (207 nm–222 nm) is strongly absorbed by proteins through peptide bonds and other biomolecules [[33], [34], [35]]. Hence, far UVC irradiation is as effective as conventional germicidal UV light in inactivating these pathogens [[36], [37], [38]].

Therefore, if UVC irradiation is used to inactivate SARS-CoV-2 in publicly occupied spaces, polymeric materials, among others, can easily be present. With these points in mind, and no comparative studies found in the literature between UVB 313 nm, UVC 254 nm, and UVC 222 nm light on the aging of polyamide 66, to provide new insights, in the present paper, controlled ultraviolet irradiation (UVB 310 nm, UVC 254 nm, far UVC 222 nm), and moisture condensation were used on polyamide 66 to evaluate the post-exposure damage comparatively. Glass transition (*Tg*) was determined along FTIR and nanoindentation since the latter is a very effective and powerful technique to evaluate the mechanical properties in small volumes of materials and thin films or surfaces of interest, such as elastic modulus (E), hardness (H), stiffness (S), and fracture toughness [39,40].

## 2 Materials and methods

### 2.1 Materials and test specimens

The polymer employed was PA 66 Vydyne 21SPF1 (Promaplast, Mexico) without photo-stabilizing additives to compare the degree of degradation caused by the three different UV lights used in the present work. A 35 Aboy saucer injection machine, using an Axxicon AIM Quick Changes mold, was used to obtain test specimens. The test piece's dimensions correspond to that specified in ISO 20753:2008 standard Type B1. Rectangular, 53 mm × 10 mm × 4 mm, samples for dynamic mechanical analysis (DMA) and FTIR were cut from these Type B1. Table 1 shows the dimensions of the type B1 test piece.

**Table 1:** Dimensions of the test pieces Type B1 according to ISO 20753:2008 standard.

Type B1	
Dimensions (mm)	
Length	80 ± 2
Width	10.0 ± 0.2
Thickness	4.0 ± 0.2

### 2.2 UV lamp characterization

In order to measure the maximum wavelength emitted by each lamp, a spectrometer. Ocean Insight Flame Miniature with a wavelength analysis range from 200 nm to 1025 nm and a resolution of 1.69 nm was coupled to an optical fiber. Lamps were placed in a dark room, and each one was turned on at a time. The spectra were recorded by a computer linked to the spectrometer, and the data were normalized.

We are interested in investigating how the action of the different wavelengths of UV irradiation degrades the samples, so the power density from UV lamps is an essential parameter in addition to ensuring that samples are illuminated with the same power per unit area. If we consider the cylindrical geometry of the UV lamps used in measurements for this work, with length *l*_*i*_ and total power P_i,_ where the subindex i indicates the i-th lamp (i = A, B or C), then if we divide the total power Pi by the area 1⁄ (2πl_i_ r) of a cylinder of length l_i and_ radius *r* we obtain the power density. Hence, the power density ρ_i_ (r) of each lamp produced at a distance *r,* can be written as:

(1) $$\rho_{i}\left(r\right)=\frac{P_{i}}{2\pi l_{i}}\frac{1}{r_{i}}$$

It is important to ensure that samples must be illuminated with the same energy density. On the other hand, it is essential to note that lamps A (313 nm), B (254 nm), and C (222 nm) have cylindrical symmetry. Hence, it is a good enough approximation to consider that UV irradiation from lamps also has cylindrical symmetry. So, the power density does not depend on angles but on r only, the perpendicular distance from the sample to the lamp. Manufacturer and model information of each the lamps A, B and C are shown in Table 2.

**Table 2:** Manufacturer and model information of each lamp A, B, and C.

Lamp	Wavelength/nm	Manufacturer	Model
A	313	QLAB	UVB-313
B	254	PHILLIPS	TUV PL-L 36W/4P 1CT/25
C	222	UVWAVE TAKE	UWM222-20W

### 2.3 UV degradation test

**Fig. 1:**
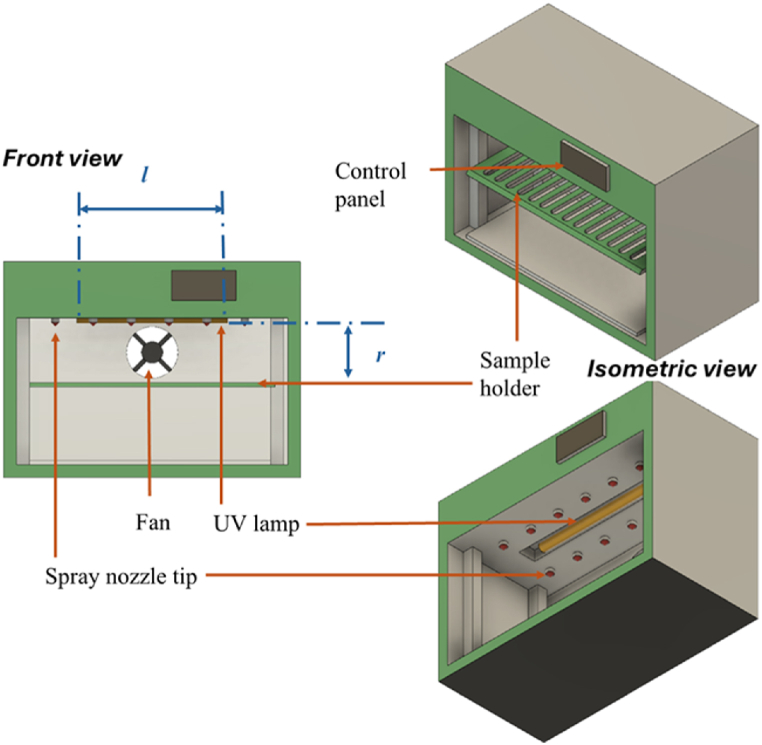
Aging chamber used for the photodegradation of polymer specimens where r stands for the distance between the sample and the lamp, and l for the lamp length.

For degradation tests, the specimens were exposed to UV-accelerated aging for 100 h, 250 h, and 500 h. For lamp B of Table 3, the samples were placed 15 cm away from the lamp, while for lamps A and C, the samples were placed 5 cm away in a rectangular UV weathering chamber 36 cm × 45 cm × 75 cm, Fig. 1. One equipped with a UVB 313 fluorescent tube emitting a maximum peak of 312 nm (40W), a second one with UVC lamps: TUV PL-L 36W/4P 1CT/25 lamp emitting a maximum peak of 253.7 nm, and a third one with a far UVC lamp 222 nm, 36 W 24V 0.8 A lamp with a spectral emission peak at 221.6 nm.

**Table 3:** Conditions at which the specimens were exposed to UV-accelerated aging for 100, 250, and 500 h.

Lamp (A) UVB 313 nm		
Treatment	T (^0^C)	Exposure time (hours)
UV irradiation	40	8
Vapor condensation	40	4
Lamp (B) UVC 254 nm		
Treatment	T (^0^C)	Exposure time (hours)
UV irradiación	40	8
Vapor condensation	40	4
Lamp (C) UVC 222 nm		
*Treatment*	*T (*^*0*^*C)*	*Exposure time (hours)*
UV irradiation	40	8
Vapor condensation	40	4

Then, 8 h of UV irradiation at 40 °C and 4 h of vapor condensation were applied, see Table 3.

In addition, to compare the interaction of UV light with PA66, the UV–Vis spectrum was obtained by diffuse reflectance using a UV–Vis spectrometer Evolution 220 (Thermo Scientific).

### 2.4 FTIR characterization

FTIR analysis was carried out using an infrared spectrometer IR Affinity 1S from Shimadzu. Spectrum was collected using an attenuated total reflectance (ATR) accessory, model Quest from Smiths, with a single bounce diamond crystal. Before the analysis, the samples were dried for 2 h at 90 °C. The surface sample exposed to UV irradiation was placed in contact with the ATR crystal, so the spectrum is obtained from the area directly affected by UV light.

### 2.5 Glass transition temperature, Tg

The glass transition temperature (*Tg)* was determined according to the ASTM E1640:2018 using a TA Instruments DMA equipment model Discovery 850. Untreated samples (not exposed to aging cycles) were conditioned to let them achieve equilibrium moisture absorption before the *Tg* measurement. For the analyses, the test pieces were exposed to a deformation amplitude of 30 μm, at a frequency of 1 Hz, and heating of 1 °C/min, for PA 66 from −30 °C to 275 °C to assess whether exposure to UV irradiation and moisture affected transitions such as melting point and glass transition temperature. These test pieces were conditioned for 5 min at the initial temperature on the equipment before starting the test.

### 2.6 Depth sensing indentation

The nano-mechanical properties were determined in an Agilent Nanoindenter model G200 equipment. The head used was the XP with a Berkovich tip with a radius of curvature R = 20 nm. Before evaluating the coatings, the nanoindenter was calibrated using a standard fused silica sample. Test parameters of area function were: C0 = 24.07, C1 = −188.17, C2 = 6872.31, C3 = −25501.41, and C5 = 17944.50. The input parameters were a maximum load of 2.5 mN, strain rate of 0.05 s^−1^ with a harmonic displacement of 1 nm and frequency of 75 Hz, and Poisson's coefficient of *ν* = 0.35, 0.36, and 0.40 for PA66. The nanoindentation tests used multiple loading cycles to evaluate the surface towards the depth at which irradiation did not affect it. In this multiple load cycle, five values of modulus of elasticity, hardness, stiffness and penetration depths are generated. The following expression defines the load in each load-unload event:

(2) $$P_{n}=\frac{nP_{\max}}{5}$$

The Oliver and Pharr method with controlled cycles was used [40,41]. The Sneddon equation calculated the stiffness as follows [42]: Where n is the event number, and $P_{\max}$ is the maximum load.

(3) $$\frac{dP}{dh}=2\beta \sqrt{\frac{A}{\pi }}E_{r}$$

Where β is a constant that depends on the geometry of the indenter (β = 1.034 for a Berkovich indenter), $E_{r}$ is the reduced elastic modulus. The elastic modulus, E was calculated by considering the compliance of the specimen and the indenter tip combined in series by the following equation:

(4) $$E=\frac{1-\nu^{2}}{\left(\frac{1}{E_{r}}-\frac{1-\nu_{i}^{2}}{E_{i}}\right)}$$

Where $E_{i}$, E and $v_{i}$, v are elastic modulus and Poisson's ratio of diamond indenter and specimen, respectively. The hardness (H) was calculated using the equation:

(5) $$H=\frac{P_{\max}}{A\left(h\right)}$$

## 3 Results and discussion

### 3.1 UV lamp characterization

**Fig. 2:**
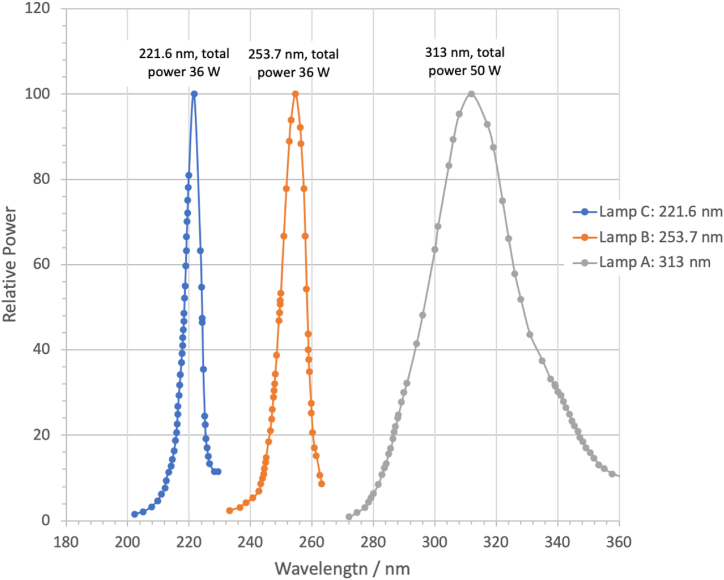
Lamp's spectrum.

A spectrometer Ocean Insight Flame Miniature was used to measure the lamp's Spectrum using a wavelength analysis range from 200 nm to 1025 nm and a resolution of 1.69 nm. Lamp's Spectrum is shown below, Fig. 2:

In Table 4, the length, power, and central wavelength of the lamps are listed.

**Table 4:** Length, power, and central wavelength of the lamps.

Lamp	Length (m)	Power (W)	Wavelength (nm)
A	0.40	40	312^a^
B	0.382	36	253.7^a^
C	0.091	36	221.6^a^

a In the present work, since the decimals are not far from integer numbers, it is decided to name them as 313 nm for lamp A, 254 nm for lamp B, and 222 nm for lamp C.

It is important to note that the lamps' spectra do not overlap. On the other hand, the power density $\rho_{i}\left(r\right)$ that a lamp *i* produces at a distance r, and considering the cylindrical symmetry of the lamps, can be written as follows:

(6) $$\rho_{i}\left(r\right)=\frac{P_{i}}{2\pi l_{i}}\frac{1}{r}$$

where $P_{i}$ is the total power of the *i* lamp, and $l_{i}$ is the length of it. Considering values shown in Table 3, the power density for lamps A and C at $r_{A}=r_{C}=r=5$ cm, is $\rho_{A}\left(r=5\;cm\right)$ ≈ 1.076 × 10^2^ W/m^2^ and $\rho_{C}\left(r=5\;cm\right)$ ≈ 1.259 × 10^2^ W/m^2^, respectively. While for lamp B, at a distance $r_{B}=15\;cm$, $\rho_{B}\left(r=15\;cm\right)$ ≈ 1 × 10^2^ W/m^2^. So, the power density of the three lamps is practically the same at these distances, i.e. $\rho_{A}\approx \rho_{B}\approx \rho_{C}\approx$ 1 × 10^2^ W/m^2^. As a result, the effect of UV irradiation on the aging of samples is analyzed at these distances. It is important to mention that, with the aim to confirm that power density remained constant during all the measurement process, the power of the three lamps A, B and C was measured after the 500 h of exposure, it was found that the power of the lamps remained without any measurable change.

### 3.2 UV degradation

**Fig. 3:**
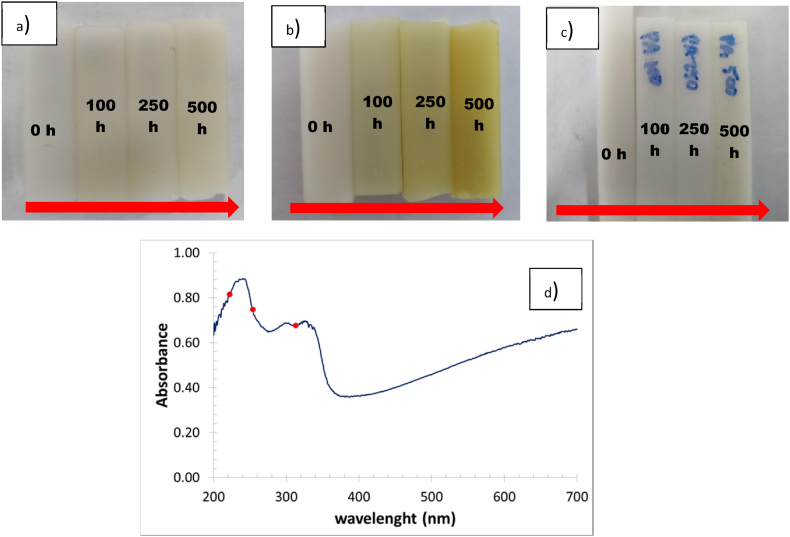
The appearance of specimens of the polyamide PA 66 after being exposed to different times (0 h, 100 h, 250 h, and 500 h) of degradation, a) UVB 313 nm, b) UVC 254 nm, and c) far UVC 222 nm, respectively, d) Absorption UV–Vis spectrum of polyamide 66 (red dots show the absorbance at the wavelengths to which the specimens were exposed).

Fig. 3 shows samples of PA 66: a) exposed to UVB irradiation at 313 nm, b) exposed to UVC irradiation at 254 nm, and c) exposed to far UVC at 222 nm. All samples show a yellowish stain product of the aging process since polyamide can absorb UV light (from 200 nm to 370 nm), as seen in Fig. 3d.

As expected, by the naked eye, it is observed that as the exposure time to radiation from UVB 313 nm, UVC 254 nm and far UVC 222 nm lamps increases, the stain on polymeric materials shows a yellowish appearance on the exposed face due to the photodegradation process. This tonality becomes more substantial at a longer exposure time. This effect is attributed to material degradation, according to Kumar et al. and Tjandraatmadja et al. [43,44]. UV radiation in the presence of ozone and some agents can accelerate the oxidation process in plastics and contribute to the degradation of the chains. In addition, Fig. 3d shows how polyamide can absorb UV light (from 200 nm to 370 nm) as highlighted by the red point corresponding to absorbance at 222, 254, and 313 nm.

### 3.3 FTIR analysis

It is well known that UV light can produce free radicals when oxygen and moisture are present [45]. When these conditions are applied to materials like polymers, free radicals can break covalent bonds leading to the formation of new ones; this effect is typically considered as damage or aging since the chemical structure of the polymer suffers a change that is appreciated as a modification in texture and yellowish or discoloration of irradiated samples. Depending on the wavelength, photon energy, time of exposition, and type of polymer, the depth of damage can range from hundreds of nanometers to hundreds of micrometers [46,47]. However, in many practical cases, it is considered surface damage. FTIR study was made with an ATR accessory to detect changes in chemical composition on the surface of polyamide samples after the aging treatment.

**Fig. 4:**
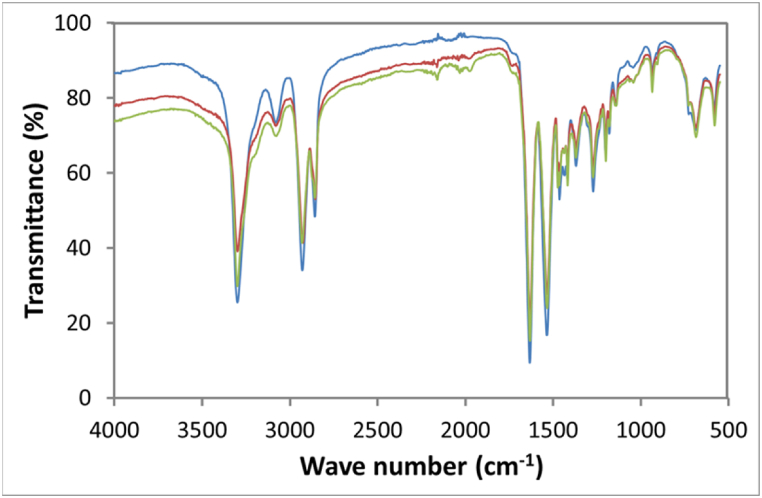
FTIR spectra of samples exposed to 313 nm UV Light during  0 h,  250 h, 500 h.

In the samples exposed to 313 nm radiation (low energy), no significant differences were observed in the spectra, as seen in Fig. 4. However, a light-yellow stain is present on the sample. Fact means that the change in functional groups (mainly amide groups) occurred to the polymer surface during aging. According to Ref. [48], the carbonyl group of the peptide linkage in polyamide materials absorbs energy at 280 nm. Absorption of this energy results in the scission of the bond between the amine group and the carbonyl group. However, it is not enough to be detected by FTIR, even after 500 h of irradiation. The fact that FTIR does not detect chemical changes does not mean that the structure of the polymer is not affected at all. Other researchers reported that PA exposed to UV aging shows a difference in mechanical behavior after aging, but no chemical changes were detected by FTIR [48].

**Fig. 5:**
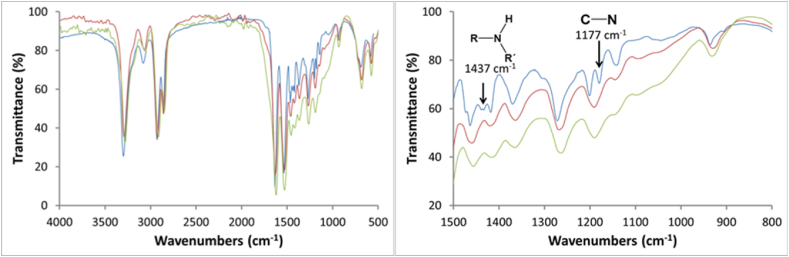
FTIR spectra (complete and zoom) of samples exposed to 222 nm UV Light during  0 h, 250 h, and  500 h.

The spectra of samples exposed to 222 nm radiation are shown in Fig. 5. It is observed that signals disappear at 1437 and 1177 cm^−1^(black arrows) in the samples that were exposed to UV light. The absorption at 1177 cm^−1^ is related to C-N stretching in secondary amines (in this case, a secondary amine), while the signals at 1437 cm^−1^ are attributed to vibrations of the amine groups, which may indicate a polymer chain break at the C-N bond.

**Fig. 6:**
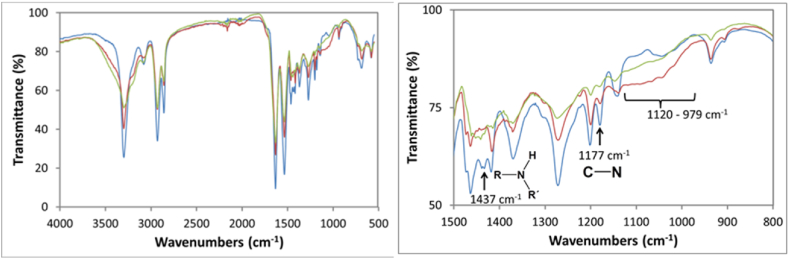
FTIR spectra (complete and zoom) of samples exposed to 254 nm UV Light during  0 h, 250 h, and  500 h.

In contrast, the samples that presented more significant evidence of change in the chemical structure of the surface affected by UV radiation were those subjected to 254 nm. In the spectra (Fig. 6), a broadening of the base of the peak is observed at 3300 cm^−1^, which indicates the presence of hydroxyl groups in the exposed samples, being more evident in the sample exposed at 500 h. On the other hand, the signals at 1440 and 1170 cm^−1^ decrease their intensity, as occurred with the samples exposed at 221.6 nm. In addition, a slightly wide and low-intensity signal begins at 1120 cm^−1^ and ends at 979 cm^−1^. This last absorption appears in the exposed samples. Although it cannot be attributed to a specific functional group or bond in the polymer, it denotes a chemical change in its structure, which could be attributed not only to chain breaking but also to crosslinking as reported in other works such as Meysam Moezzi et al. [28], Cui et al. [49] and Marek et al. [50] who found that crosslinking reactions compete with cleavage reactions in polyamides, biopolymers, and polyolefins respectively when irradiated with ultraviolet light. Ainali et al. [51] used UV 280 nm, found shifts at 1260, 1500 cm-1, and attributed them to C-N and N-H vibrations; also showed peak broadening at 3400 and attributed them to OH and NH, talked about cleavage and cross-linking reactions. Li et al. [52]aged polyamide (it is not clear which wavelength they used), the most important shift is noted between 1100 and 1300 cm-1, in the same region where we notice changes. He also sees a broadening at the base of 3400. It should be noted that none of the cited authors elucidated the polymer aging reactions; they only mention the changes in the functional groups.

### 3.4 Glass transition temperature

This analysis seeks to observe the effect that UV radiation may have on the structural behavior of the material. The $T_{g}$ value is an essential descriptor of the polymer's thermomechanical response. It is a fundamental measure of the material's propensity for mobility (between glassy to rubbery regimes of the polymer). Among the factors that enhance mobility in polymers of polar characters, such as polyamides, is the penetration of short molecules like water between the polymeric chains, which act as plasticizers lowering $T_{g}$ value [53].

**Fig. 7:**
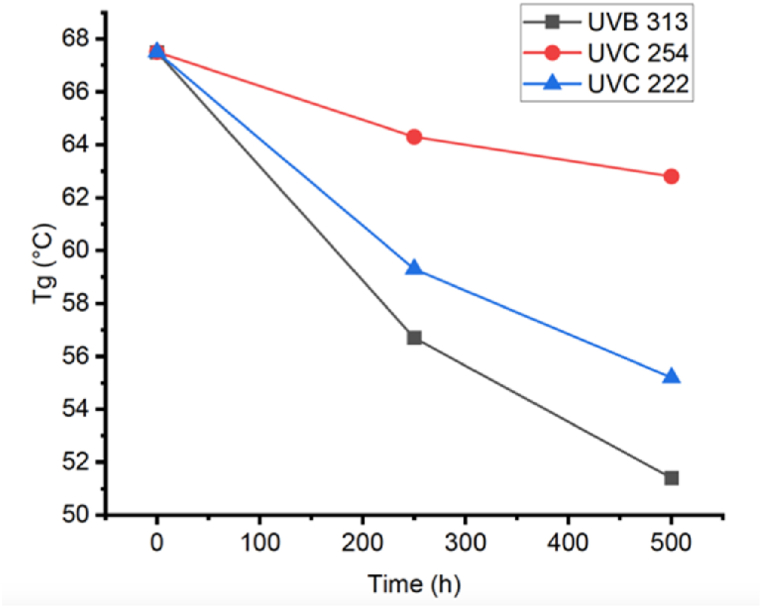
Change in glass transition temperature with time of the aging process of samples treated with different wavelengths: 313 nm, 254 nm, and 222 nm.

As described in section 2.5, the untreated samples, also referred to as samples with 0 h of aging cycles, were first conditioned to allow them to reach the equilibrium moisture absorption before $T_{g}$ was measured. The $T_{g}$ value obtained is constant over time for untreated samples since no more moisture can be absorbed or lost at equilibrium conditions, and they are not exposed to UV light that could damage the chemical structure of polymeric chains. It can be observed in Fig. 7 and Table 5 that for 0 h of treatment, the glass transition temperature was 67.5 °C. Then, a downward trend in $T_{g}$ value is seen for all cases as the time of exposure to UV light and moisture increases. Moreover, the most significant change in $T_{g}$ is shown by samples exposed to UV 313 nm after 500 h of treatment, achieving a Tg difference of 16 °C below the $T_{g}$ of untreated samples. This fact is explained as a function of a lack of sufficient surface degradation since the chemical change on the polymer surface is not enough to avoid water absorption (which may act as a plasticizer [48]. Due to this, water can reach deep inside the bulk material as exposure time increases, leading to a low $T_{g}$ value.

**Table 5:** Tg value before and after treatment with UV light and moisture.

UV Wavelength	$T_{g}$ before treatment	$T_{g}$ after 250 h of Treatment	$T_{g}$ after 500 h of treatment	$T_{g}$ difference after 500 h of treatment
313	67.5	56.79	51.45	−16.05
254	67.5	63.36	62.8	−4.7
222	67.5	59.36	55.18	−12.32

**Fig. 8:**
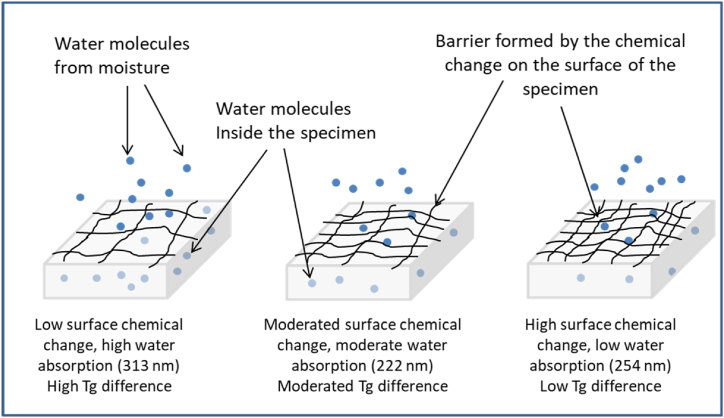
Effect of surface chemical change on water absorption and *Tg* of PA66 samples.

On the contrary, when the polymer is irradiated with a wavelength of 254 nm, significant surface degradation occurs due to scission/crosslinking reactions. As a result, a surface barrier that prevents water penetration is formed. Hence, the amount of water in the solid bulk is less in samples with significant surface chemical change, leading to a lower $T_{g}$ difference of only 4.7 °C below untreated samples. However, samples irradiated with 222 nm wavelength present moderated surface degradation, allowing moderate water permeation and showing a moderate $T_{g}$ difference (12.3 °C below untreated samples). Fig. 8 illustrates how surface aging due to UV light affects water absorption and $T_{g}$. In such a figure, the grey blocks represent PA66 specimens, dark blue circles represent water molecules from moisture, light blue circles represent water molecules inside the bulk of the specimen, and irregular black lines represent the chemical change that forms a barrier that prevents the water molecules penetration through the surface of the specimen.

### 3.5 Nanomechanical properties by nanoindentation

**Fig. 9:**
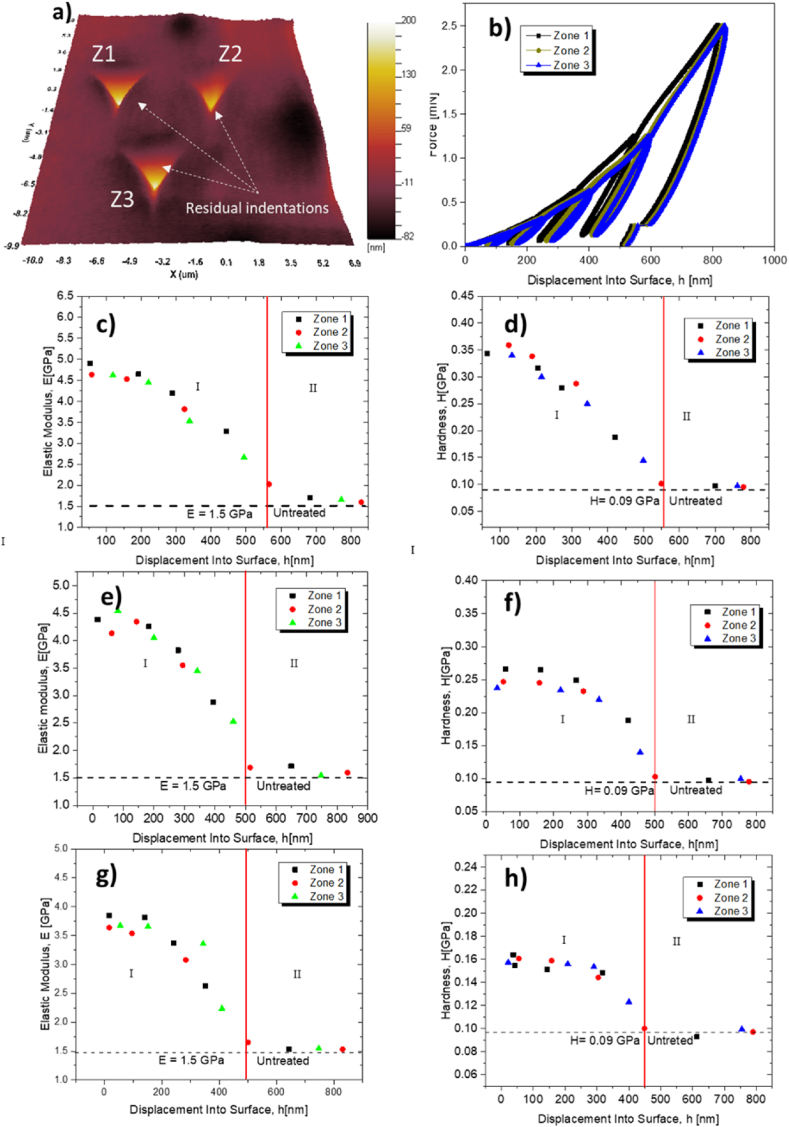
a) AFM image of residual indentations, b) illustration of load penetration depth curves in three zones in representative 254 PA66 at 500 h sample, Elastic modulus, and hardness behavior as function displacement into surface irradiation of the 254 nm, 222 nm, and 313 nm, (c–d), (e–f) and (g–h) respectively.

Sample of three areas or zones: Z1, Z2, and Z3. Fig. 9a) shows residual indentations of the Berkovich type of the three mentioned zones, which were taken in contact mode with the AFM-NanoVision attached or coupled to the Nanoindenter G200. Fig. 9b) illustrates the kind of load versus penetration depth or displacement curves into the surface, (h) curves, for a representative 254 PA 66 at 500 h of irradiation. Fig. 9c) and d) show the mechanical behavior of the elastic modulus and hardness as a function of the depth of penetration or displacement into the surface, respectively, for the 254 PA 66. These figures clearly show how the elastic modulus, E, and the hardness, H, were affected by UV radiation, increasing the elastic modulus (E = 4.76 GPa) and the hardness (H = 0.38 GPa) on the surface of the sample.

On the contrary, these properties decrease from 0 nm to 550 nm of penetration depth (h), denoted by region I. At a penetration depth between 550 nm and 800 nm, the UV radiation can no longer modify the structure since the elastic modulus and the hardness are E = 3.4 GPa, and H = 0.19 GPa, respectively, values shown by untreated sample (region II). As a result, for the 254 PA 66 sample exposed to irradiation for 500 h, the affected depth reached a penetration of h = 550 nm.

The corresponding graphs presented for the UV radiations of 222 nm and 313 nm, (e -f), and (g-h), respectively, show the same mechanical behavior described above. The elastic modulus and hardness are more significant on the affected surface and decrease as they penetrate the surface where the sample is no longer affected by such radiation. Therefore, the penetration or the depth affected by the UV radiations of 313 nm and 222 nm was h = 450 nm and h = 500 nm, respectively. Nevertheless, the penetration affected by UV radiation 313 nm is lower than the 222 nm radiation since the penetration depth was less than h = 450 nm.

**Fig. 10:**
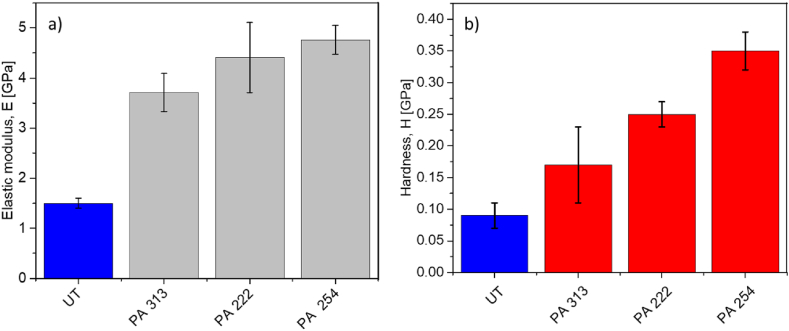
a) Elastic modulus and b) hardness on the surface of the PA 66 at different irradiations at 500 h of exposure.

On the other hand, Fig. 10 shows the mechanic behavior on the surface with respect to elastic modulus and hardness for the polymer samples, with their respective irradiation 313 nm, 222 nm, 254 nm, and the untreated UT sample at an exposure time of 500 h. We can observe how the hardness of each PA66 involved increases with respect to the irradiation used of 313 nm, 222 nm, and 254 nm, respectively, being the highest for the PA66 samples exposed to the 254 nm radiation. A fact ascribed to a cross-link that hardens the surface was described, illustrated, and discussed previously in section 3.4, Fig. 8, and according to Refs. [28,49,50]. The elastic modulus and hardness values were taken from the maximum or first values of the surface of the graphs in Fig. 9(c–h). Moreover, the same tendency observed in the FTIR results is observed for the nanoindentation results, i.e., the radiation affecting the surface's properties the most is 254 nm, while 222 nm is moderate. Therefore, the latter is recommended for cleaning and disinfection in spaces without significantly affecting these nano-mechanical properties, elastic modulus, and hardness.

## 4 Conclusions

In the present work, FTIR and nanoindentation analysis were used to detect changes in chemical composition and mechanical characteristics on the surface of polyamide samples after the aging treatment by UV light and moisture condensation. FTIR analysis showed the formation of O-H and/or N-H bonds since there appeared absorptions at 3400 cm^−1^ along with the vanishing of other signals related with C-N bonding. These changes are more evident in samples exposed to UVC 254 nm followed by samples that were irradiated with UVC 222 nm. Samples that were aged with UVA 313 nm didn't show change in FTIR spectra. In contrast, UVC 254 nm light caused the highest degree of surface chemical/mechanical damage increasing the elastic modulus (*E* = 4.76 GPa) and the hardness (*H* = 0.38 GPa) on the surface of the sample increasing the elastic modulus (*E* = 4.76 GPa) and the hardness (*H* = 0.38 GPa) on the surface of the sample, which can be attributed to cleavage/crosslinking reactions initiated by free radicals produced by UV light and moisture. Far UVC 222 nm light caused moderated affectation. On the other hand, the glass transition temperature ($T_{g}$) decreases with time after 500 h to exposure under all UV irradiation used in the present work, i.e., 51.5 °C, 55.1 °C, and 62.8 °C, respectively, vs. 67 °C for untreated samples. $T_{g}$ change is attributed to water sorbed on the polymer molecules during the treatment.

As a result, if an efficient inactivation of coronaviruses needs to be achieved, based on the results obtained, far UVC 222 nm is more likely to be used due to its moderate degradation effect on polymeric materials, but according to literature, it is effective in inactivating coronavirus.

## CRediT authorship contribution statement

**Abel Hurtado Macias:** Validation, Formal analysis. **M. Román-Aguirre:** Formal analysis. **R.P. Talamantes:** Validation. **Karen M. Soto:** Writing – review & editing, Methodology. **José Luis Reyes Araiza:** Conceptualization. **Nestor Méndez-Lozano:** Methodology. **Miguel Apátiga-Castro:** Investigation. **Jorge Pineda-Piñón:** Data curation. **José Ramon Gasca Tirado:** Validation. **José M. López-Romero:** Resources. **A. Manzano-Ramírez:** Writing – original draft, Formal analysis, Conceptualization.

## Declaration of competing interest

The authors have no competing interests to declare.
